# Effects of Probiotics on Glycemic Control and Metabolic Parameters in Gestational Diabetes Mellitus: Systematic Review and Meta-Analysis

**DOI:** 10.3390/nu15071633

**Published:** 2023-03-28

**Authors:** Enav Yefet, Liron Bar, Ido Izhaki, Rula Iskander, Manal Massalha, Johnny S. Younis, Zohar Nachum

**Affiliations:** 1 Department of Obstetrics and Gynecology, Tzafon Medical Center, Poriya 1528001, Israel; 2 Azrieli Faculty of Medicine, Bar-Ilan University, Safed 1311502, Israel; 3 Department of Evolutionary and Environmental Biology, University of Haifa, Haifa 3498838, Israel; 4 Department of Obstetrics and Gynecology, Emek Medical Center, Afula 1834111, Israel; 5 Rappaport Faculty of Medicine, Technion, Haifa 3200003, Israel

**Keywords:** glucose, insulin resistance, bifidobacterium, lactobacillus, lipid profile

## Abstract

Objectives: To assess the effects of probiotic supplements on glycemic control and metabolic parameters in women with gestational diabetes mellitus (GDM) by performing a systematic review and meta-analysis of randomized controlled trials. The primary outcome was glycemic control, i.e., serum glucose and insulin levels. Secondary outcomes were maternal weight gain, neonatal birth weight, and lipid parameters. Weighted mean difference (WMD) was used. Cochrane’s Q test of heterogeneity and *I*^2^ were used to assess heterogeneity. Results: Of the 843 papers retrieved, 14 (*n* = 854 women) met the inclusion criteria and were analyzed. When compared with placebo, women receiving probiotic supplements had significantly lower mean fasting serum glucose, fasting serum insulin, homeostatic model assessment for insulin resistance (HOMA-IR), triglycerides, total cholesterol, and VLDL levels. Decreased neonatal birth weight was witnessed in supplements containing Lactobacillus acidophilus. Conclusion: Probiotic supplements may improve glycemic control and lipid profile and reduce neonatal birth weight in women with GDM.

## 1. Introduction

Gestational diabetes mellitus (GDM) is one of the most common pregnancy complications, and its prevalence is increasing worldwide [1]. It is associated with metabolic changes, such as obesity and insulin resistance [2,3]. Uncontrolled GDM is associated with adverse pregnancy outcomes and an increased risk for long-term morbidities in both the mother and child [4,5]. Therefore, adequate glycemic control in GDM has a major role in decreasing the incidence of complications such as macrosomia, shoulder dystocia, Caesarean section, preeclampsia, GDM recurrence, and future type 2 diabetes and dislipidemia [6,7,8].

GDM treatment includes diet and lifestyle interventions, oral anti-diabetes agents, and insulin injections if needed [9]. It has been hypothesized that some of the beneficial influences of lifestyle modifications might be due to alteration of the maternal gut microbiome [10]. Various factors affect the digestive tract microbiome, including host genetics, illness, antibiotics use, diet, weight loss, and pregnancy [2,11]. Recent evidence supported an association between the gut microbiome signature and insulin metabolism in GDM [2,3,10,12].

Although appropriate treatment of GDM cannot be stressed enough, good glycemic control is difficult to achieve. Among the reasons are low compliance for lifestyle behavior changes due to poor motivation, the need for six to seven daily painful blood tests, and multiple insulin injections (around four injections) that are performed repeatedly. The adverse effects of insulin and oral hypoglycemics, such as hypoglycemia and gastrointestinal symptoms, also limited the use of those medications, and sometimes those treatments are more dangerous than hyperglycemia to the mother and fetus.

Recently, probiotic treatments were investigated in the context of metabolic diseases. Probiotics have been defined by the World Health Organization (WHO) as live microorganisms that, when taken appropriately, provide health benefits to the host [2,11,13,14]. Recently, the consumption of probiotic supplements was widely investigated for their beneficial effects on treating metabolic diseases and infections [13,15]. Intake of probiotics is a safe alternative that has shown efficacy in regulating the human gut microbial composition and function by promoting favorable metabolic activity and normalizing the gut microbiota [16,17,18]. Supplementation with probiotics has improved glycemic control and lipid profiles in patients with type 2 diabetes mellitus [17,19,20] and prevents GDM [21]. Yet, there are conflicting reports regarding its effectiveness in women with GDM [12,14,22,23,24,25,26,27,28,29,30,31]. The most common strains that were investigated to improve glycemic control in GDM were *Lactobacillus acidophilus*, *Bifidobacterium bifidum*, and *Lactobacillus casei* [12,14,22,23,25,26,27,28,29,30,31].

Our hypothesis was that probiotic supplements would improve glycemic control and lipid profile in women with GDM.

In the present study, we aimed to assess the effects of probiotic supplements on glycemic control and metabolic parameters in women with GDM by performing a systematic review and meta-analysis of randomized controlled trials.

## 2. Material and Methods

### 2.1. Eligibility Criteria, Information Sources, and Search Strategy

We performed a systematic review and meta-analysis of randomized controlled trials. The strategy search was followed the PICO (patient, intervention, comparison, and outcome) strategy. Embase, PubMed, Ovid-Medline, and Web of Science were searched using the following keywords: probiotics (probiotic/s, prebiotic/s, dietary fiber, synbiotic/s, lactobacillus, bifidobacterium, bifida) and gestational diabetes mellitus (GDM, diabetes, pregnancy/gravidarum and diabetes, pregnancy diabetes mellitus, pregnancy-induced and diabetes).

The search was restricted to English-language journals and full articles (no abstracts). All reference lists from the main reports and relevant reviews were manually searched for additional eligible publications. In addition, when clarifications were necessary or additional data were not shown in the published manuscript, the authors of the included studies were contacted. The search included all the available articles in the searched databases until 17 August 2022.

Manuscripts were included if they described a randomized controlled trial that compared probiotic treatment versus no or placebo treatment for glycemic control and metabolic parameters in women with GDM. We excluded studies on pregnant women without GDM, pregnant women with pre-GDM, studies that were not randomized controlled trials, or when there were no data on the primary outcomes.

### 2.2. Data Extraction

The credentials of the investigators were indicated in the list of authors. Two independent reviewers (LB and RI) appraised each full-text report for eligibility and extracted and tabulated all relevant data. Disagreement was settled by consensus among all authors. All procedures conformed to the guidelines for systematic review and meta-analysis of randomized controlled trial in epidemiology—PRISMA checklist [32].

### 2.3. Outcomes

The primary outcomes were levels of fasting glucose and insulin. Additional outcomes were maternal serum total cholesterol, high-density lipoproteins (HDL), LDL, very-low LDL (VLDL), triglycerides, homeostatic model assessment for insulin resistance (HOMA-IR) index, quantitative insulin-sensitivity check index (QUICKI), maternal weight gain, neonatal birth weight, large for gestational age fetus/macrosomia, and neonatal hypoglycemia. Adverse effects that might be related to the probiotics were also evaluated.

Each outcome was presented as weighted mean difference (WMD) with 95% confidence interval (CI) between the study and control groups. We performed sub-analyses in which we examined separately the effect of different strains of probiotic supplements on all the glycemic and metabolic parameters.

### 2.4. Data Synthesis and Assessment of the Risk of Bias

All reports were assigned a quality score based on the CONSORT guidelines [33]. The maximum score was 25.

Meta-analyses and review articles are exempt from the institutional review board approval in our institutions. OpenMeta Analyst software for Windows was used to perform the meta-analyses. Cochrane Q tests and the *I*^2^ (inconsistency) statistics were used to assess the heterogeneity of analyses. The random-effects model was used as a standard in determining heterogeneity between studies. The *I*^2^ values are expressed in percentages. Heterogeneity was classified as low, moderate, and high, with *I*^2^ ranging 0–25%, 25–50%, and >50%, respectively. The risk of bias was addressed by Egger’s statistics and funnel plots, and prepared using MedCalc statistical software. *p* < 0.05 was considered statistically significant.

Trial registration: This study was registered at PROSPERO (CRD42021233502).

## 3. Results

### 3.1. Study Selection

**Figure 1:**
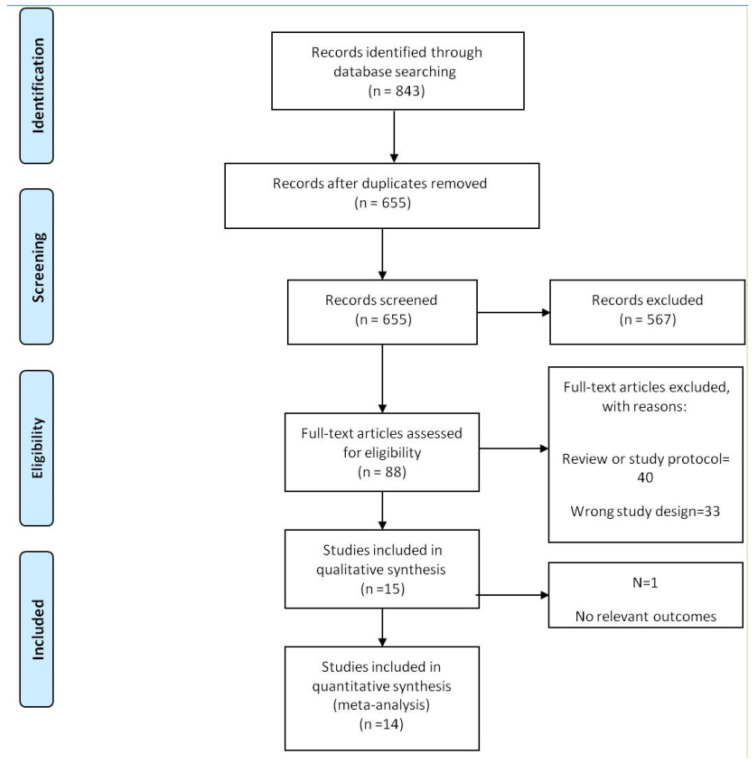
CONSORT flow of identified and appraised publications. RCT, randomized controlled trial.

The study selection process is shown in Figure 1. Of the 843 articles identified, 14 publications were deemed eligible according to the inclusion criteria [12,14,22,23,24,25,26,27,28,29,30,31,34,35].

### 3.2. Study Characteristics

Study summaries are presented in Table 1. Overall, 430 women received probiotic supplements, and 424 women were in control groups, which, in all cases, included placebo treatment. Treatment duration ranged between four weeks and until delivery. Various probiotic formulas were used. Specific adverse effects were predefined in only one study [30]. Quality scores for all studies were high. Egger’s test and a funnel plot for each outcome are presented in Table 2 and Supplement S2, respectively. Publication bias is possible in favor of the effect of probiotic towards the glucose, insulin and HOMA-IR variables according to the Egger’s test (*p* < 0.05; Table 2) and the funnel plots (Supplement S2).

### 3.3. Synthesis of Results

**Figure 2:**
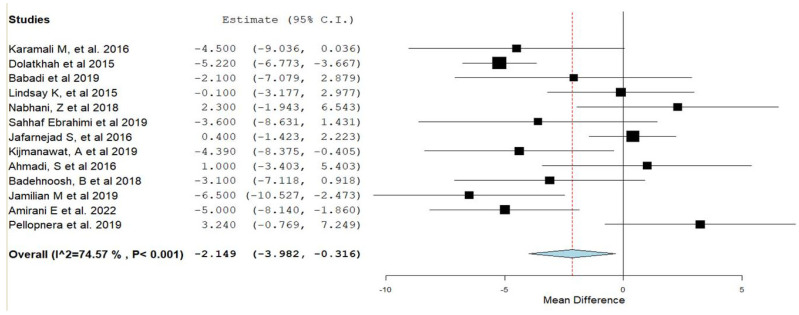
Forest plot of probiotic treatment versus placebo and fasting plasma glucose levels (mg/dL). C.I.: confidence interval [12,14,22,23,24,25,26,27,28,30,31,34,35].

**Figure 3:**
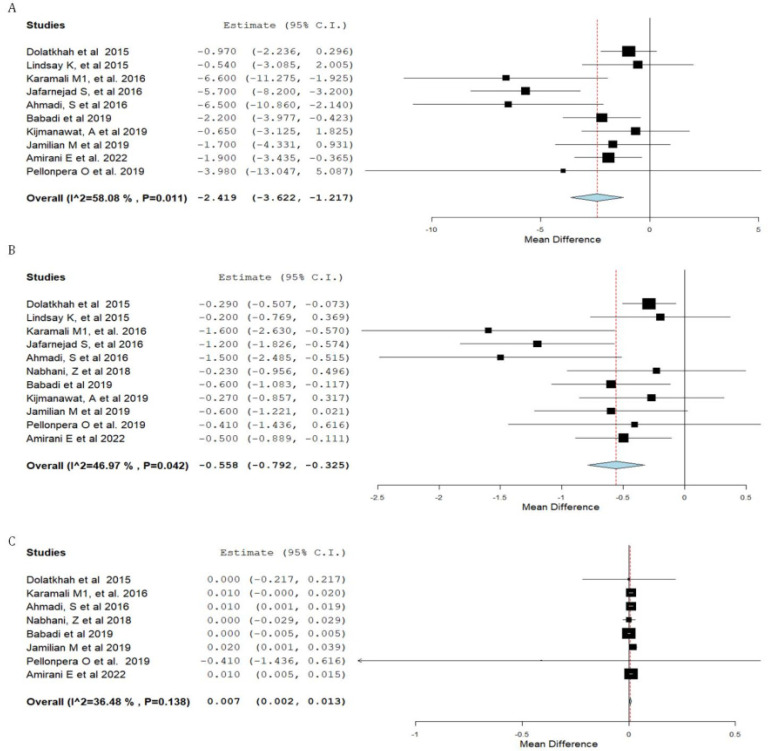
Forest plot of probiotic treatment versus placebo and insulin resistance parameters. (**A**) insulin (µIU/mL) [12,14,22,24,25,28,30,31,34,35], (**B**) homeostatic model assessment for insulin resistance (HOMA-IR) [12,14,22,24,25,26,28,30,31,34,35], and (**C**) the quantitative insulin-sensitivity check index (QUICKI). C.I.: confidence interval [12,22,25,26,28,31,34,35].

**Figure 4:**
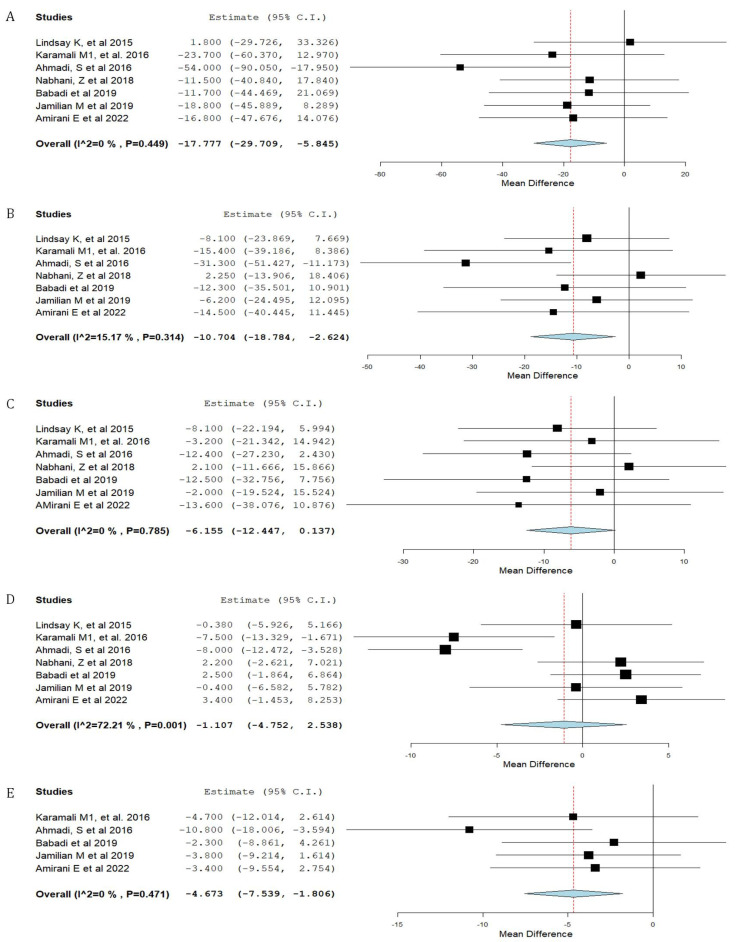
Forest plot of probiotic treatment versus placebo and lipid profile. (**A**) Triglycerides (mg/dL) [12,24,25,26,28,31,35], (**B**) total cholesterol (mg/dL) [12,24,25,26,28,31,35], (**C**) low-density lipoproteins (mg/dL) [12,24,25,26,28,31,35], (**D**) high-density lipoproteins (mg/dL) [12,24,25,26,28,31,35], and (**E**) very-low-density lipoproteins (mg/dL). C.I.: confidence interval [12,25,28,31,35].

**Figure 5:**
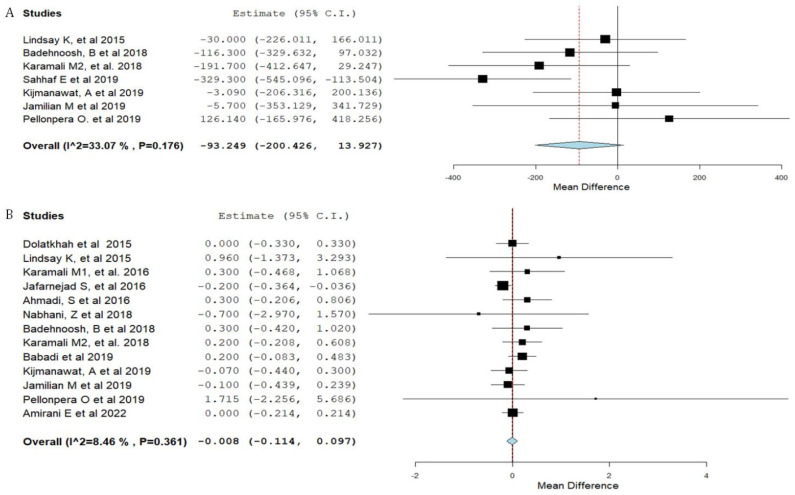
Forest plot of probiotic treatment versus placebo and neonatal birth weight (g) (**A**) and maternal weight gain (kg) [23,24,27,29,30,31,34] (**B**) C.I.: confidence interval [12,14,22,24,25,26,27,28,29,30,31,34,35].

Forest plots of the study outcomes are presented in Figure 2, Figure 3, Figure 4 and Figure 5. When compared with the placebo groups, probiotics administration was associated with a reduction in the levels of fasting plasma glucose (WMD −2.1 mg/dL 95% CI [(−4.0)–(−0.3)]; Figure 2) and fasting plasma insulin (WMD −2.4 μIU/mL 95% CI [(−3.6)–(−1.2)]; Figure 3A), HOMA-IR (WMD −0.6 95% CI [(−0.8)–(−0.3)]; Figure 3B), triglycerides (WMD −17.7 mg/dL 95% CI [(−29.7)–(−5.8)]; Figure 4A), total cholesterol (WMD −10.7 mg/dL 95% CI [(−18.8)–(−2.6)]; Figure 4B), and VLDL (WMD −4.7 mg/dL 95% CI [(−7.5)–(−1.8)]; Figure 4E), alongside higher QUICKI (Figure 3C). There was no difference between cohorts in neonatal birth weight (Figure 5A) or maternal weight gain (Figure 5B), and LDL (Figure 4C) or HDL (Figure 4D).

We performed sub-analyses in which we examined separately the effect on metabolic parameters of the three most common bacterial strains used in the probiotic formulas: *Lactobacillus acidophilus*, *Bifidobacterium bifidum*, and *Lactobacillus casei* (Table 3). All bacterial strains had favorable effects on various metabolic outcomes. *Lactobacillus acidophilus* positively affected eight parameters, including a decrease in neonatal birth weight. *Bifidobacterium bifidum* positively affected eight parameters as well and *Lactobacillus casei* positively affected five parameters (Table 3). There were no data regarding large for gestational age fetus/macrosomia and neonatal hypoglycemia. No serious adverse effects were reported for the probiotic treatment.

## 4. Discussion

### 4.1. Main Findings

In the present study, we aimed to assess the effects of probiotic supplements on glycemic control and metabolic parameters in women with GDM by performing a systematic review and meta-analysis of randomized controlled trials.

It was found that probiotic supplements improved glycemic control, insulin resistance, and lipid profile. *Lactobacillus acidophilus*, *Bifidobacterium bifidum*, and *Lactobacillus casei*, which were the most common bacterial strains used in the probiotic formulas, had favorable effects on various metabolic outcomes when assessed separately. Of interest, studies that used *Lactobacillus acidophilus* demonstrated lower neonatal birth weight in the probiotic group compared with controls.

### 4.2. Comparison with Existing Literature

GDM complicates 4–12% of pregnancies [1]. Adequate glycemic control is highly important during pregnancy, since uncontrolled GDM was associated with severe maternal and neonatal morbidities [4,5,36]. Adequate glycemic control is also important to prevent long-term maternal complications. In a mean follow-up time of 15.8 ± 5.1 years, it was found that inadequate glycemic control during pregnancies with GDM was an independent risk factor for future type 2 diabetes mellitus and dyslipidemia [7]. In addition, high post-prandial glucose levels were associated with increased risk for GDM recurrence in the next pregnancy [37].

The treatment in GDM is multidisciplinary and includes dietary and lifestyle changes such as regular exercise, as well as drug administration of oral agents or insulin in more resistant cases. Yet, those treatments are time- and effort-consuming and anti-diabetic medications have potential life-threatening adverse effects such as maternal hypoglycemia following insulin or glibenclamide use. Thus, more convenient and safe methods to treat GDM were searched for.

Probiotics have shown an efficacy in manipulating the human gut microbial composition and function to reduce the adverse metabolic effects associated with pathogenic microbial colonization [16,17]. Probiotic supplements have been shown to improve metabolism by increasing host insulin sensitivity, cholesterol metabolism, and beneficial effects on the immune system [18]. Indeed, positive effects were noticed when the use of probiotics was studied in non-pregnant individuals with diabetes mellitus [17]. Administration of *Lactobacillus acidophilus* reduced fasting glucose and hemoglobin A1C levels [19] and preserved insulin sensitivity [20] in those patients.

In pregnancy, probiotic supplements were tested in the prevention of GDM [21]. In the study of Luoto et al., the aim of the study was to determine the safety and efficacy of perinatal probiotic-supplemented dietary counseling by evaluating pregnancy outcome and fetal and infant growth during the 24-month follow-up. In total, 256 women were randomized at their first trimester of pregnancy into control and a dietary intervention groups. The intervention group received intensive dietary counseling provided by a nutritionist and were further randomized, double-blind, to receive probiotics (*Lactobacillus rhamnosus GG* and *Bifidobacterium lactis Bb12*) or placebo. The probiotic intervention significantly reduced the frequency of GDM from 34% and 36% to 13% in the control, placebo and probiotic groups, respectively. The safety of this approach was attested by the normal duration of pregnancies with no adverse events in mothers or children, and no significant differences in prenatal or postnatal growth rates among the study groups were detected [21]. In another randomized controlled clinical trial, 70 primigravida pregnant women with singleton pregnancy at their third trimester were randomly allocated to consume 200 g per day of conventional (*n* = 33) or probiotic yoghurt (*n* = 37) for 9 weeks. The probiotic yoghurt consisted of *Streptococcus thermophilus*, *Lactobacillus bulgaricus*, *Lactobacillus acidophilus LA5*, and *Bifidobacterium animalis BB12*. Fasting blood samples were taken at baseline and after a 9-week intervention to measure fasting plasma glucose and serum insulin levels. HOMA-IR was used to calculate insulin resistance score. When comparing the changes from baseline to 9 weeks of consumption of the two yogurts, the elevation in insulin resistance was milder in the probiotic group compared with the conventional group, as suggested by the lower elevation in serum insulin levels and decrease in HOMA-IR index [38]. On the contrary, in two studies that used *Lactobacillus salivarius*, no effect on glucose metabolism was noticed, neither in obese pregnant woman [39] nor women with GDM [24]. Notably, both studies used *Lactobacillus salivarius*, while the studies that demonstrated a positive effect used different strains. In addition, low gastric and intestinal motility in pregnancy might require a higher dose of probiotic to achieve an effect.

In our study, sub-analysis of different probiotic strains yielded different metabolic effects. These results stress the fact that probiotic supplements are a heterogenic group consisting of various bacteria, and each one can act differently on glucose and metabolic pathways. In future studies there should be more focus on the effect of each bacterial strain in order to characterize the appropriate supplement for each metabolic disorder. Such a strategy will be able to achieve a more robust effect while avoiding false-negative results that can be found following the integration of various kinds of probiotic supplements.

In pregnant women, the intestinal bacterial composition has been implicated in alterations in insulin, c-peptide, HOMA-IR, and hemoglobin A1C levels, as well as low-grade inflammatory responses, which lead to GDM manifestations [40,41,42]. Maternal insulin resistance leading to hyperglycemia and fetal hyperinsulinemia has been suggested to underlie fetal overgrowth and macrosomia [43] and increased maternal lipid levels regardless of glycemic control [44].

Probiotic supplements were suggested to improve glucose, insulin, and lipid metabolism and decrease inflammatory response, reducing the risk for GDM and unfavorable pregnancy outcomes [45,46]. The mechanisms by which probiotic supplements alter glucose metabolism include the production of short-chain fatty acids, which were found to (1) regulate the production of hormones such as leptin and grehlin [47], affecting energy intake and expenditure, (2) increase the intestinal expression of peptide YY and glucagon-like peptide-1 (GLP-1) hormones, which act to increase insulin sensitivity [48], and (3) enhance the production of glucagon-like peptide-2 (GLP-2), which reduces inflammation [49,50]. Probiotic administration in women with GDM was also reported to reduce inflammatory markers, such as high-sensitivity C-reactive protein, tumor necrosis factor-α, and interleukin-6 [14,50].

### 4.3. Strengths, Limitations and Suggestions

The strengths of this meta-analysis lay in its incorporation of 14 high-quality, randomized, placebo-controlled trials with a large sample size of 854 women, as well as the investigation of various metabolic parameters that are known to affect pregnancy outcomes in GDM. Its limitations included inter-study heterogeneity concerning the type of probiotic supplement, the effective dose range and duration of treatment, and a possible publication bias toward the positive effect of the probiotic supplements on GDM parameters. Another limitation was that only fasting glucose was evaluated but not mean daily glucose or postprandial glucose levels, which were demonstrated to better predict pregnancy complications in GDM [36]. Future studies should examine the effect of probiotic supplements on the daily glucose charts, including pre-prandial, postprandial, and mean daily glucose values, since those are used to evaluate glycemic control and respond to treatment in clinical settings. Neonatal outcomes that are associated with GDM should also be evaluated in a more comprehensive manner.

## 5. Conclusions

Probiotic supplements may improve glycemic control and lipid profile and reduce neonatal birth weight in women with GDM.

## Figures and Tables

**Table 1:** Summary of included studies.

First Author, Year	Country	Number of Subjects	Probiotic Intervention	Probiotic Dose/Day	Intervention Period	Primary Outcome	Results	Quality Score *
Dolatkhah et al., 2015 [22]	Turkey	Probiotics *n* = 29 placebo *n* = 27	*Lactobacillus acidophilus* LA-5, *Bifidobacterium* BB-12, *Streptococcus thermophilus* STY-31 and *Lactobacillus delbrueckii bulgaricus* LBY-27	4 biocap >4 × 10^9^ CFU	8 weeks	Weight gain and glucose metabolism	Decrease in FPG	24
Lindsay et al., 2015 [24]	Ireland	Probiotics *n* = 48 placebo *n* = 52	*Lactobacillus salivarius* UCC118	100 mg of Lactobacillus salivarius UCC118 at a target dose of 10^9^ CFU	8 weeks	Fasting glucose	No impact on glycemic control among GDM patients	25
Karamali et al., 2016 [12]	Iran	Probiotics *n* = 30 Placebo *n* = 30	*L. acidophilus*, *L. casei* and *B. bifidum* strains	2 × 10^9^ CFU/g each	6 weeks	Glucose homoeostasis parameters	Decrease in FPG and serum insulin levels	25
Jafarnejad et al., 2016 [14]	Iran	Probiotics *n* = 37 Placebo *n* = 35	VSL#3 (*Streptococcus thermophilus*, *Bifidobacterium breve*, *Bifidobacterium longum*, *Bifidobacterium infantis*, *Lactobacillus acidophilus*, *Lactobacillus plantarum*, *Lactobacillus paracasei*, and *Lactobacillus delbrueckii* subsp. *bulgaricus*)	112.5 × 10^9^ CFU	8 weeks	Glycemic control and inflammatory status	FPG, HbA1c, HOMA-IR, and insulin levels remained unchanged	22
Ahmadi et al., 2016 [25]	Iran	Probiotics *n* = 35 Placebo *n* = 35	*Lactobacillus acidophilus*, *Lactobacillus casei*, *Bifidobacterium bifidum* plus 0.8 g inulin	2 × 10^9^ colony-forming units/g each	6 weeks	Insulin metabolism	Decrease in serum insulin levels	25
Nabhani et al., 2018 [26]	Iran	Probiotics *n* = 45 Placebo *n* = 45	*Lactobacillus acidophilus*, *Lactobacillus plantarum*, *Lactobacillus fermentum*, *Lactobacillus gasseri*	500 mg of *Lactobacillus probiotic* strains consisting of *L. acidophilus* (5 × 10^10^ CFU/g), *L. plantarum* (1.5 × 10^10^ CFU/g), *L. fermentum* (7 × 10^9^ CFU/g), *L. gasseri* (2 × 10^10^ CFU/g)	6 weeks	Glucose homoeostasis parameters	No effect on FPG and insulin resistance/sensitivity indices	25
Badehnoosh et al., 2018 [27]	Iran	Probiotics *n* = 30 Placebo *n* = 30	*Lactobacillus acidophilus*, *Lactobacillus casei* and *Bifidobacterium bifidum*	2 × 10^9^ CFU/g each	6 weeks	Inflammatory markers	Decrease in FPG, no effect on pregnancy outcomes	24
Karamali et al., 2018 [29]	Iran	Probiotics *n* = 30 Placebo *n* = 30	*Lactobacillus acidophilus* (2 × 10^9^ CFU/g), *Lactobacillus casei* (2 × 10^9^ CFU/g) and *Bifidobacterium bifidum* (2 × 10^9^ CFU/g) strains plus 800 mg inulin	2 × 10^9^ CFU/g each	6 weeks	Inflammatory markers	No effect on birth weight	23
Babadi et al., 2019 [28]	Iran	Probiotics *n* = 24 Placebo *n* = 24	*Lactobacillus acidophilus*, *Lactobacillus casei*, *Bifidobacterium bifidum*, and *Lactobacillus fermentum*	2 × 10^9^ CFU/g each	6 weeks	Gene expression of PPAR-γ	Decrease in FPG, serum insulin levels and insulin resistance; increased insulin sensitivity	23
Sahhaf Ebrahimi et al., 2019 [23]	Iran	Probiotics *n* = 42 Placebo *n* = 42	Probiotic yoghurt containing *Lactobacillus acidophilus* and *Bifidobacterium lactis*	300 g/day of probiotic yoghurt (contained 10^6^ *Lactobacillus acidophilus* and *Bifidobacterium lactis*	8 weeks	Glycemic parameters	Decrease in FPG and HbA1c, lower birth weight and fewer macrosome neonates in the probiotic group	24
Kijmanawat et al., 2019 [30]	Thailand	Probiotics *n* = 28 Placebo *n* = 29	*Lactobacillus acidophilus* and *Bifidobacterium bifidum*	1 × 10^9^ CFU/g each	4 weeks	Glycemic control	Decrease in FPG and serum insulin levels, and increased insulin sensitivity	25
Jamilian et al., 2019 [31]	Iran	Probiotics *n* = 29 Placebo *n* = 28	*Lactobacillus acidophilus*, *Bifidobacterium bifidum*, *Lactobacillus reuteri*, and *Lactobacillus fermentum*	8 × 10^9^ CFU/day	6 weeks	Insulin metabolism	Decrease in FPG and serum insulin levels	25
Pellonpera et al., 2019 [34]	Finland	Probiotics *n* = 27 Placebo *n* = 22	*Lactobacillus* *rhamnosus* and *Bifidobacterium* *animalis* ssp. *lactis*	1 × 10^10^ CFU each	From the first study visit, throughout the pregnancy, and until 6 months postpartum	The incidence of GDM	No difference in FPG, insulin resistance, maternal weight gain and neonatal birth weight	24
Amirani et al., 2022 [35]	Iran	Probiotics *n* = 26 Placebo *n* = 25	*Lactobacillus acidophilus*, *Bifidobacterium bifidum*, *Bifidobacterium lactis Bifidobacterium longum* Additionally, selenium	2 × 10^9^ CFU/day each	6 weeks	Insulin metabolism	Reduced fasting glucose, insulin concentrations, insulin resistance, triglycerides, total cholesterol, and low-density lipoprotein (LDL) cholesterol	24

* Quality scores were based on the CONSORT checklist. The maximum score was 25. Abbreviations: CFU, colony-forming units; GDM, gestational diabetes mellitus, FPG, fasting plasma glucose; PPAR-γ, peroxisome proliferator-activated receptor gamma.

**Table 2:** Egger’s test for asymmetry.

Outcome	*p* Value
FPG	0.03
Fasting plasma Insulin	0.048
Neonatal birth weight	0.95
HOMA-IR	0.005
QUICKI	0.57
Triglycerides	0.24
VLDL cholesterol	0.052
Total cholesterol	0.37
LDL cholesterol	0.43
HDL cholesterol	0.96
Maternal weight gain	0.93

Abbreviations: FPG, fasting plasma glucose; HOMA-IR, homeostatic model assessment for insulin resistance; HDL, high-density lipoproteins; LDL, low-density lipoproteins; QUICKI, the quantitative insulin sensitivity check index; VLDL, very-low-density lipoproteins.

**Table 3:** Sub-analysis of metabolic effects according to specific bacteria.

	FPG (mg/dL)	Fasting Insulin (µIU/mL)	Neonatal Birth Weight (g)	HOMA-IR	QUICKI	TG (mg/dL)	VLDL (mg/dL)	Total Cholesterol (mg/dL)
*Lactobacillus acidophilus*	**−2.8** **[(−4.7)–(−0.9)]**	**−2.7** **[(−4.0)–(−1.3)]**	**−141** **[(−262)–(−19)]**	**−0.6** **[(−0.9)–(−0.4)]**	**0.008** **[0.002–0.01]**	**−21** **[(−34)–(−8)]**	**−4.7** **[(−7.5)–(−1.8)]**	**−12** **[(−22)–(−1.7)]**
*Bifidobacterium* *bifidum*	**−3.7** **[(−5.5)–(−2.0)]**	**−2.4** **[(−3.8)–(−1.1)]**	−88 [(−204)–(27)]	**−0.7** **[(−1.0)–(−0.4)]**	**0.008** **[0.002–0.015]**	**−23** **[(−38)–(−9)]**	**−4.7** **[(−7.5)–(−1.8)]**	**−16** **[(−26)–(−6)]**
*Lactobacillus casei*	−2.2 [(−4.5)–(0.1)]	**−4.5** **[(−7.9)–(−1.2)]**	−153 [(−306)–(1)]	**−1.1** **[(−1.6)–(−0.6)]**	0.006 [(−0.002)–0.013]	**−29** **[(−54)–(−4)]**	**−5.8** **[(−10.8)–(−0.8)]**	**−21** **[(−34)–(−8)]**
	**Maternal Weight Gain (Kg)**			**HDL (mg/dL)**		**LDL (mg/dL)**		
*Lactobacillus acidophilus*	−0.006 [(−0.1)–(0.1)]			−1.2 [(−5.5)–(3.0)]		−5.7 [(−12.7)–(1.4)]		
*Bifidobacterium* *bifidum*	0.072 [(−0.05)–(0.2)]			−1.9 [(−6.9)–(3.0)]		**−8.4 [(−16.6)–(−0.2)]**		
*Lactobacillus casei*	0.16 [(−0.01)–(0.34)]			−4.2 [(−11.3)–(2.9)]		−9.6 [(−19.6)–(0.4)]		

Values are presented as weighted mean difference (95% confidence interval). Statistically significant values are marked in **bold**. Abbreviations: FPG, fasting plasma glucose; HOMA-IR, homeostatic model assessment for insulin resistance; HDL, high-density lipoproteins; LDL, low-density lipoproteins; QUICKI, the quantitative insulin sensitivity check index; TG, triglycerides; VLDL, very-low-density lipoproteins.

## Data Availability

No new data were created in this meta-analysis.

## Supplementary Materials

The following supporting information can be downloaded at: https://www.mdpi.com/article/10.3390/nu15071633/s1.
